# On the Continued Fevers of Great Britain

**Published:** 1840-03-07

**Authors:** George C. Shattuck

**Affiliations:** Boston


### MEDICAL EXAMINER.

DEVOTED TO MEDICINE, SURGERY, AND THE COLLATERAL SCIENCES.

No. 10.] PHILADELPHIA, SATURDAY, MARCH 7, 1840. [Vol. III.

### ON THE CONTINUED FEVERS OF GREAT BRITAIN.

By George C. Shattuck, Jr., M. D., of Boston.

(Concluded.)

### THIRD OBSERVATION.

A widow, æt. forty-one, a seamstress, was brought to the hospital the 21st of February, 1839. Her hair was of a chesnut colour, her complexion fair, her muscular system slightly developed. Her general health was good; she had never had any long illness. She had always lived at London, and recently had nursed a sister who had been received at the hospital as a fever patient.

The 14th of February she was perfectly well; she slept but little the night of the 14th and 15th. The morning of the 15th, headach, pains of the limbs, ringing in the ears, diminished appetite; she continued to work, but with difficulty. In the evening, chills and shivering. No dejections the first few days; she took a powder, the administration of which was followed by two liquid stools. Since the 18th she has been confined to her bed, and since the same date she has eaten nothing. Sleep recently disturbed by dreams. No epistaxis.

February 22d, the condition of the patient was as follows:—The countenance uniformly red, natural; the decubitus dorsal; the memory exact; the answers correct; headach less severe than at commencement; dizziness; no ringing in the ears; the hearing acute; the eyes sensible to the light, but neither red nor suffused. Quiet during the night; little sleep; no delirium. The tongue whitish, moist; anorexia; thirst. The abdomen somewhat developed, sonorous, tender on strong pressure, gargouillement; the spleen cannot be felt below the ribs. One liquid stool since entrance. On the abdomen were four or five rose spots, slightly raised above the surface of the skin, disappearing on pressure. No sudamina. The respiration accelerated, easy, equal on both sides, without râle. Pulse one hundred, moderately full; heat moderate.

The common febrifuge of the hospital, and two drachms of castor oil, were prescribed. The next day the oil was vomited, and twelve drops of the wine of colchicum, were added to each spoonful of the febrifuge. Gruel for drink.

*Feb. 25th.*—The countenance pale, heavy, stupid expression. Talked much in night; somnolence; some muttering; easily awakened; answers correct; exercise of mental faculties difficult; no headach; eyes neither red nor suffused; hearing acute. Tongue yellowish; its central part dry. The abdomen developed, sonorous, tender on strong pressure. Two liquid stools. Four or five lentricular rose spots on the abdomen; other smaller spots grouped together, disappearing on pressure, not raised above the surface. The two forms of eruption run into each other. Pulse one hundred and ten, slightly developed; skin hot, dry.

*March 1st.*—Violent delirium.

*March 2d.*—Stools and urine involuntary.

*March 4th.*—Countenance dull; somnolence; eyes neither red nor suffused; patient does not answer questions. Pulse one hundred and four, small. Decubitus on the right side almost abdominal; stools and urine involuntary; agitation in the night; she talked a good deal.

Can any one doubt that this patient had typhoid fever? The headach, the ringing in the ears, the dizziness, the sleep disturbed by dreams, the meteorism, the tenderness of the abdomen, the diarrhœa, the rose spots, all these are symptoms of typhoid fever; and yet we have here the eruption which, but for the fifth observation, we might have been disposed to look upon as characteristic of another form of fever.

Sudamina were found in one case of the first series.

*Anatomy.*—There being but one fatal case of the first series, and the observation having been given, it is unnecessary to repeat the anatomical details, so that it now remains to examine the four fatal cases of the second series.

*Small intestine.*—In one case the small intestine was contracted, in the others its size was natural. Its contents were composed of bile and mucus. In one case the mucous membrane of the ileum was of a rose tint. The glands of Peyer were barely visible in two of the cases. The thickness and consistence of the mucous membrane were natural in all the cases.

*Large intestine.*—In one case the large intestine was contracted. In the same case, there was redness and softening of the mucous membrane of the cœcum and descending colon. In one of the cases the mucous membrane of the cœcum was of a deep red colour; in a third case, that of the cœcum and first third of the colon were softened; in the fourth case, there was nothing remarkable in the large intestine. In all the cases the fæcal matter was small in quantity, pultaceous, soft, yellowish.

*Stomach.*—In two cases the mucous membrane of the stomach was natural. In one of these cases the organ was distended with gas.

In one case the membrane was mamelonated and softened in the space of an inch and a half square, near the cardiac orifice. In the fourth case the membrane was injected in points, and softened in the great extremity and along the great curvature; towards the pyloric orifice it was mamelonated and thickened.

*Mesenteric glands, liver, spleen.*—The mesenteric glands were natural in all the cases. In one case the liver contained rather more blood than usual. The quantity of bile in the gall-bladder was from one to two ounces. This fluid was black and viscous in three subjects, thin and yellowish in a fourth. The spleen was black and very pliable in one case, and not remarkable in the others. There was nothing remarkable about the genito-urinary apparatus in any case.

*Organs of respiration.*—In three of the cases old adhesions of the lungs, and recent adhesions in a fourth case. The lower lobe of the right lung of this last subject was grayish, firm, heavy, not crepitating, friable, reduced into pulp on pressure, its section granulated. The lower half of the middle lobe of the same lung was in a similar condition. Elsewhere the lung was grayish, light, crepitated well. The difference between the healthy and the diseased tissue was marked. On the left side the posterior part of the lower lobe was deprived of air; its tissue was red, granulated, friable, and at one point, in the extent of an inch, the colour was grayish, its other characters remaining. In the superior lobe, at one inch from the summit, was a hard nucleus; and when this was cut into, a purulent infiltration was found, in the centre of which was a little fluid pus. In this case we must acknowledge the existence of a pleuro-pneumonia; but, from the history of the case during life, we must consider it a secondary affection. In two of the other cases the lower lobes of the right lung were of a violet colour, friable, not granulated, a great quantity of a reddish liquid flowing out on cutting into them. In all the cases the bronchial mucous membrane was injected and lined by a considerable quantity of mucus.

*Organs of circulation.*—The volume of the heart was not increased in any case; its consistence was natural, and in one patient it was florid. There were from one to two ounces of black, liquid blood, in the right ventricle. The left ventricle contained somewhat less in three cases, and was empty in a fourth. Clots were found in the right ventricle in all the cases, and three times in the left ventricle. They were small and fibrinous in three subjects,—blackish in the subject with pneumonia. The lining membrane, the valves, the pericardium, were natural in all the cases. The aorta had a slight rose tint, in patches, in one case. The veins were not remarkable, even in one subject where a sanious effusion was found in the wrist and elbow joints.

*Head.*—Notwithstanding the violent delirium in many cases, the organs in the cranial cavity presented no remarkable lesions, so that we must consider the delirium a functional lesion. This accords with the results of M. Louis. In three cases there was a slight sub-arachnoid infiltration. In two cases two spoonfuls of clear serum were found in each of the lateral ventricles. The pia mater, in one case, was moderately injected. No alteration of consistence or colour was found in the brain, in the cerebellum, or in the medulla oblongata.

*Skin, cellular tissue, joints.*—In three cases the eruption was found after death. The spots penetrated the thickness of the skin to the subcutaneous cellular tissue, and communicated to the parts they occupied a purplish colour. In one patient, the wrist and elbow joints contained from one to two ounces each of a sanious fluid. The synovial membrane of both joints was smooth, and of a slight rose colour. The veins of the arm contained only black blood.

*Exterior of the body.*—In no case was there a marked emaciation. The cadaverous rigidity was well marked in all. Stripes on the back were noted in the four cases. In two of the cases the autopsy took place eight and ten hours after death, the weather being cold and damp; and, in two other cases, twenty and twenty-five hours had elapsed, the weather being the same. The lesions that have been described can hardly be attributed to commencing putrefaction.

*Treatment.*—We cannot come to any positive conclusions as to the influence of different methods of treatment, inasmuch as no two patients were treated exactly alike.

Stimulants, as sherry wine and brandy, were given to four patients of the second class. In three cases the disease was not arrested, became more severe, and at last terminated fatally. In the other case the treatment was commenced at the beginning of the fourth week; and this patient took in the first eight days sixteen ounces of brandy, forty-two ounces of wine, and four quarts of beef tea. This patient was convalescent towards the middle of the fifth week. One of the patients of the first series took four ounces of wine and a pint of beef tea daily from the eleventh day, but the disease went on towards a fatal termination.

Purgatives and laxatives were given in several cases, but no modification of the disease appeared to follow their administration.

We have not the means of comparing susceptibility to treatment in the cases of the two series; but, from the analysis of the symptoms and lesions, we learn that the conclusions which we might have been disposed to allow from a consideration of the two first cases, must be modified. Three observations remain, and perhaps no better disposition can be made of them than to submit them as they were taken.

FOURTH OBSERVATION.

Mary Beach, aged 28, was received at the London fever hospital the 15th of February, 1839. She has always lived in London, and for some years has been employed in a brush manufactory. Her hair of a chesnut colour, her eyes gray, her muscular system moderately developed; she has always enjoyed good health.

The 8th of February she was taken with chills, headach, pain in the back and limbs. The chills continued during two or three hours, and were followed by heat, thirst, inappetence, loss of strength, so that she was obliged to keep her bed. No diarrhœa before February 11th, when she took some castor oil and some powders.

February 16th, the ninth day of her disease, her condition was as follows: The countenance pale, expression of stupor, of prostration, and some anxiety. The answers slow but exact, the exercise of the mind difficult, the eyes neither red nor suffused, the hearing acute. She was quiet in the night, yet slept little.

The tongue was badly protruded, dry; the teeth encrusted; the abdomen somewhat developed, sonorous. The patient complained more when pressure was made there, than when other parts of the body were touched. One stool of a natural colour and consistence, and two liquid stools after taking two drachms of castor oil. The respiration accelerated, equal on the two sides, free from râle. The patient sat up in bed with some difficulty. On the abdomen and chest were numerous spots of a deep red colour, of a variable size, grouped together, imperfectly disappearing on pressure. The spots on the arms were less numerous but of the same character.

℞.—Sherry wine ℥iv; beef tea, one pint. Tea for drink.

*Feb.* 17.—No headach, some stupor, little sleep in night, though the patient was quiet; the tongue brownish and dry, the abdomen well formed, painful on pressure; no gargouillement, two stools.

The wine and beef tea continued.

*Feb.* 18.—On waking from sleep in the night, four or five times she screamed and was agitated. The eyes red, suffused; the cheeks red, stupor, prostration; the patient scarcely answers questions. The central part of the tongue dry, its edges moist. The pulse 120, small; the abdomen supple, tender on pressure. One involuntary stool.

Hydrarg. cum. creta gr. v, Pulv. ipec. comp. gr. iij, every four hours.

*Feb.* 19.—Drowsiness, the eyes moist, red; the pulse 112, small; the tongue dry, badly protruded: the abdomen well formed, not tender on pressure; one liquid stool.

*Feb.* 20.—The prostration marked, two stools.

*Feb.* 22.—The countenance more natural, the eyes still red and suffused; the patient answered more naturally; the central part of the tongue dry, its edges moist; the abdomen well formed; one liquid stool. The pulse 96, regular; the eruption less distinct than on the preceding days.

*Feb.* 25.—The pulse 80, the heat moderate, the patient has two ounces of bread and two thirds of a quart of beef tea.

We may perhaps consider this case as belonging rather to the second series, and yet we have meteorism, pain of the abdomen on pressure, and diarrhœa. The following case resembles, in some points, the last.

FIFTH OBSERVATION.

A huckster, aged 21, with chesnut hair, hazel eyes, a light complexion, and of rather a delicate constitution, was admitted into the London fever hospital the 21st of February, 1839. He was born and has always lived in London. His general health good; the 17th of February he was seized with chills, shivering, headach, diminished appetite, ringing in the ears, imperfect and scanty sleep at night. Some medicine was administered, and followed by liquid stools. He walked to the hospital, though with considerable difficulty.

*Feb.* 25.—The pulse 120, small; the heat moderate, moist; the answers correct, no headach, quiet through the night. The countenance natural; the eyes neither red nor suffused; the sight clear; the hearing acute. The central part of the tongue dry, its edges moist; thirst, anorexia; the abdomen natural, not painful on pressure; two or three liquid stools since taking oil.

The fever mixture was prescribed that day, and two drachms of castor oil in the morning. On the 26th, the patient took six ounces of wine, and eight ounces on the 27th.

*Feb.* 28.—No delirium, scarcely any sleep during the night; the countenance natural, the answers correct; no headach, no dizziness, no ringing in the ears; the tongue tremulous, its middle part whitish, its edges moist; thirst, anorexia; the abdomen well formed, not painful on pressure; gargouillement; the spleen is not felt below the ribs. The patient sits up easily. Lenticular rose spots, and other spots smaller, of a fainter red, imperfectly disappearing on pressure, grouped together.

*March* 4.—Some of the small faint spots may still be discovered in the abdomen. The patient walks in the ward.

In this case the abdominal symptoms are wanting, but we have the two eruptions. In the following case, the difficulty of diagnosis is greater.

SIXTH OBSERVATION.

Mary Reckett, a widow, and mother of three children, 36 years of age, of middle height, moderate embonpoint, chesnut hair and blue eyes, has lived at London for the last eighteen years, and for the last two years has been a laundress in the London fever hospital. Her health has been uniformly good since that time, her habits regular, her diet generous.

The 31st of January she was quite well, and was employed in washing the clothes of the porter of the hospital, who had just died of fever. On the 1st of February, she was taken with chills, headach, pain in the back and limbs.

The same day her appetite was diminished by one half, she had some colics, and two or three liquid stools. She slept but little that night, continued to work with great discomfort to herself, that day and the next; her symptoms continued, and Feb. 4th she was obliged to give up work, and took possession of a bed in the wards of the hospital.

The 7th of February, and the seventh day of her illness, she was in the following condition: She was lying on her back, her countenance pale and anxious, her eyes neither red nor suffused; her mind was sufficiently clear, her memory distinct; she complained of pain shooting through her head, from the temples to the occiput; no dizziness, the sight clear, the hearing natural, no ringing of the ears. She had not bled at the nose. Weakness, inability to sit up; she had slept quietly in the night, two hours at a time. No pain in the back or limbs.

The tongue was white, moist; the thirst intense, anorexia; for four days she had eaten nothing. The form of the abdomen was natural; it was rather sonorous, painful on pressure, perhaps more so in the right iliac fossa. The spleen could not be felt; she had had three liquid stools in the last twenty-four hours. No rose spots, no sudamina; the respiration slightly accelerated, dry and unfrequent cough: the resonance of the chest was natural, the respiration equally well heard on both sides, free from râle. The day before, a sharp pain along the last ribs of the right side yielded immediately to a blister applied in that region. Nothing unnatural could be discovered in the præcordial region; the pulse moderately develoged, 90. The temperature somewhat increased, moist.

℞.—Ten ounces of blood to be taken from the temples by cupping glasses; gruel for drink.

*Feb.* 8.—The pain of the head diminished immediately after the cupping, and at present she scarcely suffers there at all. Two lenticular rose spots, disappearing on pressure, were found in the right side of the abdomen; three liquid stools. In other respects, the condition of the patient was the same as on the day before. Towards evening she had an ill turn, feeling faint, for about half an hour. She slept scarcely at all in the night.

*Feb.* 9.—No headach; she sat up in her bed easily, and did not become dizzy.

The pulse was 100, sufficiently strong; she had five or six liquid stools in the last twenty-four hours, and four or five rose spots were discovered on the abdomen. No gargouillement. An effervescent draught was prescribed, and she was allowed to take gruel.

*Feb.* 10.—The pulse was 112, the skin hot and dry; she had been quiet in the night, no delirium; she complained when pressure was made on the abdomen; three liquid stools. There were three or four rose spots on the abdomen and sudamina just above the clavicles. The prescription of the day before was continued. On the 12th of February, and the twelfth day of the disease, the prostration was marked, the mind clear, the pulse 120, the central part of the tongue dry; the abdomen painful on pressure, the patient complained also when her limbs were touched. One liquid stool; two or three rose spots.

*Feb.* 13.—Several small round spots of a deep red colour, scarcely disappearing on pressure, not raised above the skin, were discovered on the chest and abdomen.

*Feb.* 14.—The eyes were injected and suffused; the patient was anxious; no stupor, no headach, no sleep during the night; the sight clear, the hearing acute; the eruption more general than the day before, over body and limbs; the pulse 124, the central part of the tongue yellowish; three stools; the patient complained when pressure was made on the abdomen, the chest, or on the limbs. She sat up in bed with difficulty. The respiration without râle.

Hydrarg. cum. creta gr. iij, ipec. comp. gr. iij, every six hours. Six ounces of sherry wine.

The next day, the 15th, the patient was delirious, agitated, and it was difficult to obtain any answers to questions; the pulse 108, small and feeble, the respiration 24; the tongue moist, the abdomen somewhat swollen, the patient complained when pressure was made on it.

She had passed no urine for several hours, and a considerable quantity was drawn off by the catheter. The prescription of the day before was continued. The following day the prostration was greater; the patient continued to be delirious, though not violently so. Five ounces of brandy, six grains of camphor, and three of ammonia were prescribed.

*Feb.* 17.—The eruption was as on the preceding days; the pulse 150, small and feeble; three involuntary stools. Death took place on the morning of the 18th day.

After six days of mild and damp weather, the abdomen was opened, its contents removed, and carefully examined. The stomach of a moderate size, distended with gas, and containing scarcely any liquid. Its mucous membrane violet in the great cul de sac, grayish elsewhere, softened in the great cul de sac and along the great curvature, so that its consistence was that of mucus. The intestine much distended by gas. The small intestine contained a yellowish liquid, its mucous membrane softened throughout so as to give no strips; its more dependent parts of a violet colour.

Peyer's patches and the mesenteric and mesocolic glands were natural. At two inches from the ilea cœcal valve the ileum was about four inches in circumference, the cœcum six inches. The mucous membrane of the large intestine was in the same condition as that of the small intestine. No trace of ulceration could be discovered in it. The spleen was eight inches long, its greatest thickness sixteen lines, its greatest width three inches and four lines, its substance yielding very easily to the pressure of the finger.

The other viscera were natural.

Of what disease did this patient die? The diarrhœa, the tenderness of the abdomen on pressure, the meteorism, the colics at the commencement, may lead us to suspect that there must have been some inflammation of the mucous membrane of the intestine, and it is to be regretted that the degree of decomposition at the time of the autopsy was such, that we cannot be sure what was the condition of the intestinal canal at the moment of death. We must also regret that there was no opportunity to examine the thoracic viscera and the brain. Still, imperfect as was the autopsy, one thing results from it, that the lesion of Peyer's patches, the anatomical character of the typhoid fever of Paris, was absent. And yet considering the history of the patient during life, can we hesitate in calling the disease typhoid fever? The abdominal symptoms are more marked than in many patients in whose intestines the disease of Peyer's patches has been found. And if it be said that dizziness and dimness of vision were wanting, it should be remembered, that these symptoms, though usual, are not always present in typhoid fever, and that they were noted as frequent symptoms in the epidemic described by Dr. Gerhard as typhus. And then the cerebral symptoms of this patient are not those which Dr. Gerhard has given us as distinctive of the epidemic of Philadelphia from the typhoid fever of Louis. The general sensibility of the skin is not often met with in typhoid fever, and the eruption of this patient is not exactly such as is usually found in that disease. The rose spots were present, but they were followed by an abundant petechial eruption. Certainly, then, there are some peculiarities in this case, yet inasmuch as the symptoms of the typhoid fever are not always identical, but vary within certain limits, I cannot see in the history of the patient during life, any reason for refusing to consider the case one of typhoid fever, and though from this case we cannot say that continued fever is one disease, and that the lesion of Peyer's patches is comparatively unimportant, and by no means essential, we do see that the question is more difficult than a perusal of the two first observations would have led us to suppose, and we are reminded how necessary are numerous facts for the solution of important pathological questions.

### *Remarks on the Memoir of Dr. Shattuck.*

BY W. W. GERHARD, M. D.

The observations upon which the memoir of Dr. Shattuck is founded, were communicated by him to the Medical Society of Observation of Paris. These cases were analyzed and published by Dr. Valleix, one of the editors of the Archives of Medicine. I am induced to publish the concluding remarks of Dr. Valleix's paper, although they are perhaps of greater interest to me, than to most of my readers. They refer to the distinction between the two forms of fevers; this has, it is true, been often hinted at, but that it has never been pointed out clearly, is sufficiently proven by the confusion still existing in the works of most writers upon fever. The observations upon which the memoirs published by me in the American Journal, (February and August, 1837,) were founded, were derived from an epidemic of typhus, which prevailed in certain parts of Philadelphia, from March, 1836, to about the same period in 1838; at times subsiding for a little while, but not entirely ceasing. The cases upon which the memoirs were founded, occurred in 1836; that is when the epidemic tendency of the disease was best characterized. In the year 1837, a large number of cases were admitted from the passenger ships, which were filled with Irish emigrants; in one of them the disease was traceable, by direct succession, to a patient who was clandestinely introduced on board of one of them at Liverpool. We had, therefore, direct evidence that these cases, which differed from those of our epidemic only in degree, were the genuine British typhus. They were quite mild, and the mortality was insignificant. These cases were compared with cases of typhoid fever at the same time, and in my clinical lectures I have repeatedly pointed out the difference of symptoms in patients labouring under the two diseases, and placed side by side. With careful observation I believe that the two affections may be always, or nearly always, distinguished; at least I have never, I believe, been mistaken when I have known the course of symptoms, and but rarely, when I have seen the patients even for a day or two.

The emigrants arriving in this country, are not so often affected with true typhus, whether of the mild or severe form, as with typhoid fever, which is much more frequent, and sometimes extends to a considerable number of the passengers, assuming an evidently infectious character. But when typhus does exist, the number affected in each ship is much greater, as the disease, under such circumstances, is transmitted from one to another with great facility.

The following are the concluding remarks in Dr. Valleix's analysis of Dr. Shattuck's cases.

"The greater number of authors who have treated of this subject before Dr. Gerhard of Philadelphia, have regarded the two diseases in question as altogether identical, and not finding in the typhus the lesions of the glands of Peyer, they have regarded them as neither constant nor characteristic. I do not intend to enter upon this debated question, which I have treated in detail in my article of last February, published in this journal, to which I refer the reader. I will merely mention that the researches of all these writers have been incomplete and altogether insufficient. But I will examine with more care, the memoir of Dr. Gerhard, which is, without contradiction, the most important of those which have been published on typhus fever. The eruption in the patients observed by this author, was as well characterized as in the patients whose cases I have published, but it was not exactly the same with respect to the abdominal and cerebral symptoms. The first were much less frequent and much lighter in the typhus of Philadelphia. But this is only a difference of degree, which may be explained by the very exciting treatment followed at London. As to the symptoms furnished by the organs of the senses, such as the troubled sight, ringing in the ears, deafness, Dr. Gerhard found them more intense and more frequent than in typhoid fever; in the English patients it was quite different. Is not this difference owing to Dr. Gerhard having observed during the course of an epidemic, in which all the symptoms might well be more severe than in sporadic cases? This explanation is admissible. But before receiving it, we must remark that Dr. Gerhard has not made an exact comparative examination of typhus and typhoid fever, in cases taken under the same circumstances, and that some error may have crept into his approximative estimate. In every other respect, the results of his researches entirely agree with those which I have already stated, and have led this observer to the same conclusion, which is necessarily deduced from the preceding facts; that is, that typhus fever and typhoid fever are distinct diseases.

"But the present memoir had another object; it was to show that cases taken in England, offered us examples of both affections, and that consequently typhoid fever is the same in all countries, where continued fever has been observed. It was therefore an error with the English physicians, to treat as one single and identical affection, all the fevers which they met with, and unless they seek to arrive at a more certain diagnosis, they will never attain a truly useful therapeutic result. We may hope that more extended researches than those which have served as a basis to this memoir, will finally clear up so important a question."

